# Probiotics for treating acute diarrhea in children: an evidence synthesis

**DOI:** 10.3389/fped.2025.1722257

**Published:** 2026-01-12

**Authors:** Cheng Chen, Pei Liu, Lang Xiao, Qian Cao, Ding'an Zhou, Xingmei Liu, Min Shen, Xu Jia, Lin Zhang

**Affiliations:** 1 School of Basic Medical Sciences, Chengdu Medical College, Chengdu, Sichuan, China; 2 Key Laboratory of Microbial Drugs Innovation and Transformation, Yan'an University, Yan'an, Shaanxi, China; 3 Life Science and Engineering, Southwest Jiaotong University, Chengdu, Sichuan, China; 4 Clinical Research Center, Affiliated Hospital of Guizhou Medical University, Guiyang, Guizhou, China; 5 Non-coding RNA and Drug Discovery Key Laboratory of Sichuan Province, Chengdu Medical College, Chengdu, Sichuan, China; 6 Department of Pharmacy, Shaoxing People’s Hospital; Shaoxing Hospital, Zhejiang University School of Medicine, Shaoxing, Zhejiang, China

**Keywords:** acute diarrhea, children, meta-analyses, nutrition, probiotics

## Abstract

**Objective:**

We aimed to evaluate the efficacy of probiotics against diarrhea in children.

**Background:**

Acute diarrhea remains the leading cause of childhood morbidity and mortality worldwide and is one of the most common reasons for child visits and hospitalizations.

**Methods:**

Randomized clinical trials (RCTs) of probiotics for the treatment of acute diarrhea in children were identified by searching EMBASE, PubMed, the Cochrane Library, and the Clinicaltrials.gov registry with a search deadline of September 29, 2023. Fixed- and random-effects models were employed, using standardized mean differences (SMDs), relative risk ratios (RRs) and 95% confidence intervals (CIs) as outcome indicators. Primary outcomes included the duration of the child's diarrhea and the number of children with diarrhea at the completion of the trial, and secondary outcomes included the length of the child's hospitalization, the frequency of stools on day 2 post-intervention, and the number of recoveries within 3 days of the intervention. We performed this according to the Preferred Reporting Items for Systematic Reviews and Meta-Analysis (PRISMA) statement.

**Results:**

The study included 25 randomized controlled trials involving 9,071 subjects. After the intervention, moderate evidence indicated that, probiotics shortened the duration of diarrhea in children (SMD=−0.44, 95% CI: −0.70∼−0.17), increased the number of children who recovered from diarrhea, reduced the number of children with diarrhea at the completion of the trial (RR = 0.68, 95% CI: 0.54∼0.86), and reduced the number of stools on d 2 (SMD=−0.38, 95% CI: −0.59∼−0.18), but the quality of evidence was very low. However, there was no effect on children's length of hospitalization (SMD=−0.27, 95% CI: −0.63∼0.09) and the number of recoveries within 3 d after the intervention (RR = 1.70, 95% CI: 0.98∼2.97). Among these, probiotics reduced the average duration of diarrhea by approximately 1.21 days. For the primary outcome, subgroup analyses based on individual probiotic strains, *Limosilactobacillus reuteri* was found to have a significant trend in reducing the duration of diarrhea (SMD=−0.62,95% CI:−0.92∼−0.32), while *Lacticaseibacillus rhamnosus* was found to have a significant trend in decreasing the number of children with diarrhea at the completion of the trial (RR = 0.52, 95% CI:0.37∼0.74).

**Conclusion:**

The results showed that probiotics demonstrated adequate clinical efficacy in shortening the duration of diarrhea, increasing the number of recoveries in children with diarrhea, reducing the number of diarrhea cases, and alleviating diarrhea symptoms.

**Systematic Review Registration:**

https://www.crd.york.ac.uk/PROSPERO/, PROSPERO CRD42024534039.

## 1 Introduction

Acute gastroenteritis is one of the most common diseases affecting children worldwide and typically manifests as diarrhea, vomiting, and abdominal pain. Acute diarrhea is common in infants and remains the leading cause of childhood morbidity and mortality worldwide (1). Viral, bacterial and parasitic intestinal infections are the most common causes of acute diarrhea in children and are associated with poor sanitation, poor personal hygiene and unsafe water supply (2); other important causes of acute diarrhea in children include antibiotics, infections not related to the gastrointestinal tract, food poisoning and allergies (3).

Probiotics are defined as “live microorganisms that confer a health benefit to the host,” primarily by enhancing gut barrier function and restoring intestinal flora balance. Synbiotics, on the other hand, consist of probiotics combined with nondigestible dietary fibers that selectively stimulate the growth and activity of certain colonic microorganisms, thereby benefiting host health (4, 5). Probiotics have been shown to improve gut health, alleviate symptoms associated with lactose intolerance, and reduce the risk of diseases such as inflammatory bowel disease, infectious diarrhea, and allergies (6, 7). Several meta-analyses have showed that probiotics may improve the prognosis of children with acute gastroenteritis through several mechanisms, including promoting intestinal microflora balance, boosting host immunity, and enhancing the gut barrier function (8–12). However, the trials included in these studies had methodological limitations, including small sample sizes, unclear randomization strategies, inadequate concealment of treatment allocation, and lack of sufficient evidence for the efficacy of probiotics in the treatment of gastroenteritis and other indications (8). Two trials conducted in Canada and the United States have questioned the efficacy of probiotic strains in the treatment of children with acute gastroenteritis (8, 13, 14). There is uncertainty about the efficacy of probiotics and inconsistent recommendations for their use, and despite evidence supporting the use of specific probiotics in certain clinical situations, further research is needed to confirm their effectiveness (13).

This study aimed to collect, critically evaluate, and systematically analyze published randomized controlled trials on probiotics for treating diarrhea. The goal was to provide a basis for the clinical use of probiotics and to offer evidence-informed guidance for the prevention and treatment of childhood diarrhea.

## 2 Materials and methods

### 2.1 Study design and ethical considerations

This study was conducted under the Preferred Reporting Items for Systematic Reviews and Meta-Analysis (PRISMA) statement (15); this article reports the results of the literature search and does not involve any animal, cellular, or human experimental studies. This study did not require ethical approval in China.

### 2.2 Data sources and search strategy

Two authors identified studies through September 29, 2023, using the terms probiotics, diarrhea, and children in EMBASE, PubMed, the Cochrane Library database, and the Clinicaltrials.gov registry. The search strategy is detailed in Supplementary Appendix S1.

### 2.3 Selection criteria

Two researchers screened the literature and reviewed the title and abstract of each paper.

Inclusion criteria:

1. Randomized controlled trials
2. Children (under 18 years of age)
3. Acute diarrhea
4. Studies written in English
5. The patient was diagnosed with diarrhea and had to report at least one diarrhea outcome.
6. Use of any strain of probiotic, compared to placebo or no treatment

The exclusion criteria were nonrandomized trials, studies of malnourished children, studies with no children, studies with no relevant outcomes, studies with no probiotics, studies with no diarrhea, case reports, reviews, meta-analysis studies, conference abstracts, animal studies, *in vitro* experiments, letters, and studies where data and full text were unavailable by various methods.

### 2.4 Data extraction

Two researchers independently extracted data from each included article. The following characteristics of the trials were collected: authors, year of publication, study site, age, intervention, and sample size. The outcome indicators included duration of diarrhea, number of children with diarrhea at the completion of the trial, length of hospitalization, frequency of stools on d 2, and number of recoveries within 3 d of the intervention.

### 2.5 Risk of bias assessment

Two researchers independently assessed the risk of bias for each trial using the Cochrane Risk of Bias 2 Tool (ROB2). Five domains were evaluated: bias during randomization, bias in deviating from established interventions, bias in missing outcome data, bias in outcome measurement, and bias in reporting outcome selection. The risk of bias for each area can be categorized into three levels: “low risk of bias”, “some concerns,” and “high risk of bias”.

### 2.6 Evidence quality assessment

Two investigators independently assessed certainty of evidence for each outcome, with discrepancies resolved by a third reviewer. Certainty of evidence was evaluated using the Grading of Recommendations Assessment, Development, and Evaluation (GRADE) framework, which divides evidence into very low, low, moderate, and high levels (16). According to the GRADE approach, the certainty of the evidence of RCT was initially considered as high. However, it may be downgraded due to five factors (risk of bias, inconsistency, indirectness, imprecision, and publication bias).

Risk of bias: Evidence quality should be downgraded if most studies exhibit high risk of bias in one or more critical domains. Inconsistency: Evidence quality should be downgraded if substantial unexplained heterogeneity exists among study results. Indirectness: Evidence quality should be downgraded if any lack of directness exists in the population, intervention, comparator, or outcome (PICO), based on whether the evidence directly addresses the current PICO question. Imprecision: Evidence quality should be downgraded if results are imprecise, such as insufficient sample size or overly broad confidence intervals. Publication bias: Evidence quality should be downgraded if publication bias is suspected.

### 2.7 Statistical analysis

Analyses were performed using R language statistical software version 4.04. The data are expressed as the mean ± standard deviation, continuous outcome variables were assessed using the SMD and 95% CI, and dichotomous outcomes were assessed using the RR and 95% CI. Statistical heterogeneity was assessed with the *χ*2 test, and the degree of heterogeneity among the studies was measured by the I^2^ statistic. An I^2^ greater than 50% indicated significant heterogeneity and a random-effects model was applied; a fixed-effects model was applied when there was no heterogeneity between studies (17, 18).

## 3 Results

### 3.1 Study selection and research flow chart

**Figure 1:**
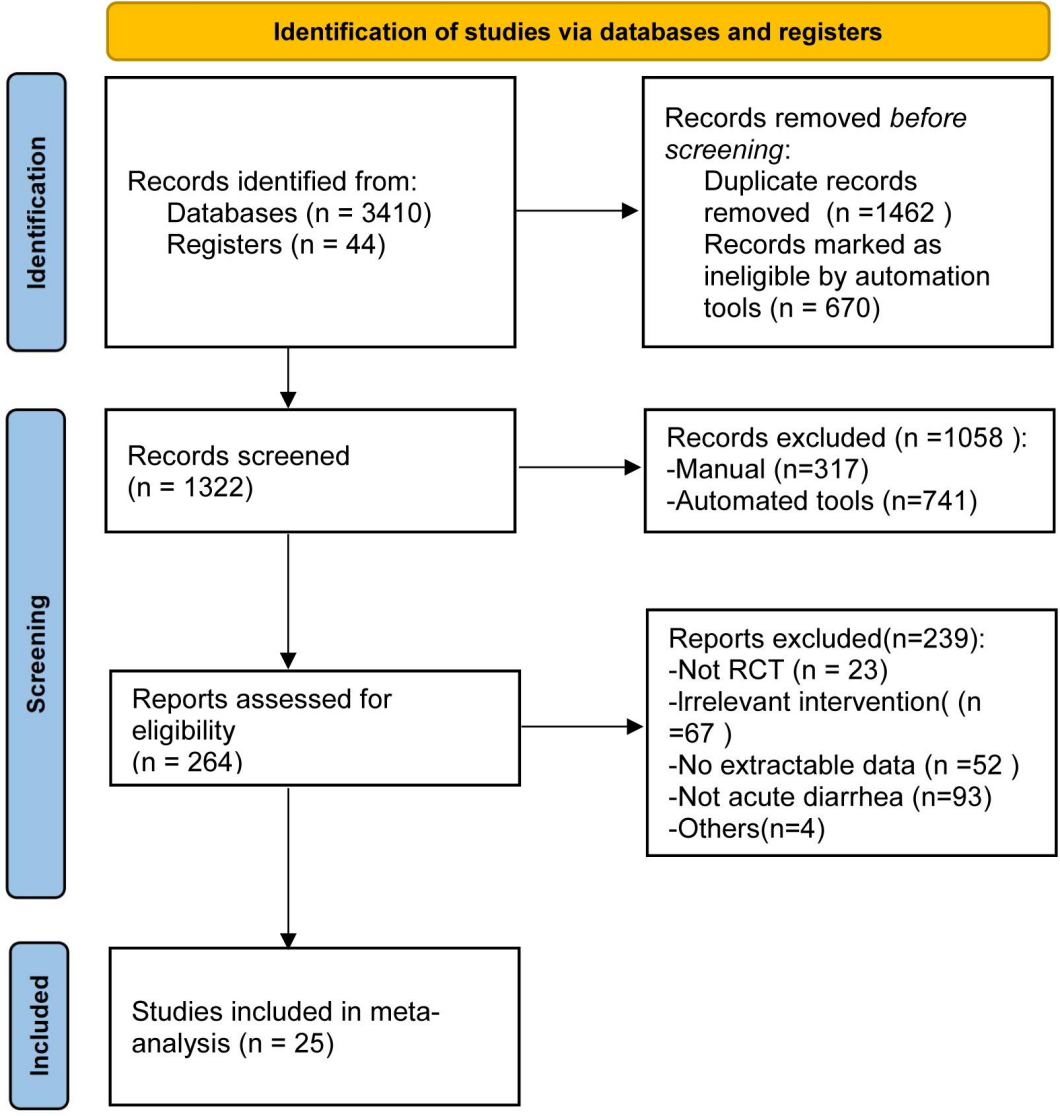
Flowchart for selection of studies on the efficacy of probiotics on diarrhea in children.

To determine the efficacy of probiotics for the treatment of diarrhea in children, 3,554 records were retrieved from EMBASE, PubMed, the Cochrane Library database, and the Clinicaltrials.gov registry. After removing duplicates, 264 potentially eligible articles were identified. Ultimately, 25 randomized controlled trials that met the inclusion criteria were included in our study (Figure 1).

### 3.2 Baseline characteristics of the included studies

Our study included 25 RCTs involving 9,071 subjects that were published between 2004 and 2023 (8, 15, 19–41). The baseline characteristics included study, study site, age, intervention, sample size, and relevant outcome indicators (Table 1).

**Table 1:** Baseline characteristics of the Included Studies Evaluating Probiotics for the Treatment of Acute Diarrhea in Children.

Study ID	Study	study site	Intervention	Age	N	Number of children with diarrhea at the completion of the trial	Number of recoveries within 3 d of the intervention	Duration of diarrhea (day)	Duration of hospitalization(day)	Frequency of stool on day 2	Dosage(CFU/D)	Daily dosing frequency	Treatment duration(day)
1	Lukasik J 2022	Netherlands	Multispecies probiotic	50 ± 52.25	158	33	NA	5 ± 2.99	NA	NA	1.0 × 10^10^	1	7–17
			Placebo	50 ± 52.25	155	50	NA	4.7 ± 2.99	NA	NA			
2	Schnadower D 2018	United States	Lacticaseibacillus rhamnosus	18.48 ± 12.47	468	NA	NA	2.15 ± 2.10	NA	NA	2.0 × 10^10^	2	5
			Placebo	18.48 ± 12.47	475	NA	NA	2.29 ± 1.96	NA	NA			
3	Freedman SB 2018	Canada	Lacticaseibacillus rhamnosus and Lactobacillus helveticus	16.6 ± 10.90	414	NA	NA	2.32 ± 2.40	NA	NA	8.0 × 10^9^	2	5
			Placebo	16.6 ± 10.90	413	NA	NA	2.48 ± 2.54	NA	NA			
4	Sur D 2010	India	Probiotic	35.5 ± 13.57	1,802	608	NA	NA	NA	NA	6.5 × 10^9^	1	84
			Control	35.5 ± 13.57	1,783	674	NA	NA	NA	NA			
5	Kluijfhout S 2020	Egypt	Multispecies probiotic	58.87 ± 107.33	26	NA	NA	3.04 ± 1.36	NA	NA	6.5 × 10^9^	1	7
			Placebo	58.87 ± 107.33	20	NA	NA	4.20 ± 1.34	NA	NA			
6	Lai HH 2019	China	Lacticaseibacillus rhamnosus	27.9 ± 15.6	42	8	NA	NA	NA	NA	4.0 × 10^8^	2	7
			Control	27.6 ± 16.1	39	16	NA	NA	NA	NA			
7	Kosek MN 2019	Peru	Limosilactobacillus reuteri	3.81 ± 1.26	41	8	NA	NA	NA	NA	1.0 × 10^8^	1	5
			Placebo	3.81 ± 1.26	19	6	NA	NA	NA	NA			
8	Dinleyici EC 2015	Turkey	Limosilactobacillus reuteri	27.9 ± 18.2	29	13	NA	2.52 ± 1.02	NA	NA	1.0 × 10^8^	1	5
			Control	22.6 ± 14.4	31	27	NA	3.1 ± 0.64	NA	NA			
9	Maragkoudaki M 2018	Greece	Limosilactobacillus reuteri	1.7 ± 0.7	28	NA	NA	1.6 ± 0.63	NA	NA	1.0 × 10^9^	1	5
			Control	1.8 ± 0.7	23	NA	NA	2.06 ± 1.02	NA	NA			
10	Mai TT 2021	Vietnam	Lacticaseibacillus casei	51.7 ± 10.0	510	25	NA	4.8 ± 7.0	NA	NA	6.5 × 10^9^	1	7
			Control	54.1 ± 8.6	493	39	NA	3.9 ± 5.0	NA	NA			
11	Kołodziej M 2019	Poland	Limosilactobacillus reuteri	25.7 ± 35.2	123	25	NA	NA	NA	NA	2.0 × 10^8^	2	10
			Placebo	25.8 ± 33.8	124	16	NA	NA	NA	NA			
12	Bruzzese E 2016	Italy	Lacticaseibacillus rhamnosus	33.8 ± 17.4	45	2	NA	NA	3.9 ± 1.6	NA	6.0 × 10^9^	2	15
			Placebo	34.7 ± 17.2	45	11	NA	NA	4.9 ± 1.2	NA			
13	Sindhu KN 2014	India	Lacticaseibacillus rhamnosus	14.05 ± 6.81	64	18	NA	NA	NA	NA	1.0 × 10^10^	1	28
			Placebo	14.05 ± 6.81	59	25	NA	NA	NA	NA			
14	Aggarwal S 2014	India	Lacticaseibacillus rhamnosus	19.18 ± 12.78	100	NA	NA	2.59 ± 0.56	NA	NA	1.0 × 10^10^	1	5
			Control	20.02 ± 14.02	100	NA	NA	3.34 ± 0.56	NA	NA			
15	Mourey F 2020	India	Saccharomyces boulardii	13.2 ± 8.1	49	NA	40	2.74 ± 0.5	NA	3.1 ± 0.9	1.0 × 10^10^	2	5
			Placebo	13.3 ± 8.2	51	NA	8	3.97 ± 0.73	NA	3.6 ± 1.3			
16	Hong Chau TT 2018	Vietnam	Lactobacillus acidophilus	16.27 ± 7.11	150	10	NA	1.72 ± 1.5	3.29 ± 1.56	NA	4.0 × 10^8^	2	5
			Placebo	16.55 ± 6.74	150	11	NA	1.72 ± 1.5	3.26 ± 1.59	NA			
17	Salazar-Lindo E 2004	Peru	Lacticaseibacillus casei	14.9 ± 7.5	82	NA	52	2.44 ± 1.26	3.38 ± 1.36	NA	1.0 × 10^9^	1	5
			Placebo	14.7 ± 6.4	78	NA	51	2.1 ± 1.17	3.11 ± 1.4	NA			
18	Passariello A 2012	Italy	Probiotic	20.46 ± 12.25	52	NA	35	3.77 ± 1.86	NA	2.4 ± 1.61	2.5 × 10^9^	1	5
			Placebo	20.46 ± 12.25	55	NA	22	4.58 ± 2.12	NA	3.3 ± 1.85			
19	Dutta P 2011	India	Bacillus coagulans	12 ± 4	78	NA	70	1.42 ± 0.85	NA	NA	2.4 × 10^8^	2	5
			Placebo	11 ± 4	70	NA	58	1.52 ± 0.89	NA	NA			
20	Vandenplas Y 2011	Belgium	Multispecies probiotic	47.66 ± 37.46	57	NA	NA	3 ± 1.52	NA	3 ± 1.52	6.5 × 10^9^	1	7
			Placebo	47.66 ± 37.46	54	NA	NA	4.35 ± 0.76	NA	3.65 ± 2.28			
21	Szymanski H 2006	Poland	Lacticaseibacillus rhamnosus	24.6 ± 17.7	46	NA	NA	3.48 ± 2.32	NA	NA	2.4 × 10^10^	2	5
			Placebo	26.8 ± 20.8	41	NA	NA	4 ± 2.98	NA	NA			
22	Ruszczynski M 2008	Poland	Lacticaseibacillus rhamnosus	54.8 ± 45.3	120	9	NA	4.4 ± 2.2	NA	NA	4.0 × 10^10^	2	14
			Placebo	53.5 ± 44.25	120	20	NA	4.1 ± 2.1	NA	NA			
23	Teran CG 2009	Bolivian	Multispecies probiotic	6.9 ± 3.3	25	NA	NA	2.38 ± 1.06	3.46 ± 1.4	6.79 ± 7.08	2.5 × 10^9^	2	5
			Placebo	11 ± 5.2	25	NA	NA	3.11 ± 1.11	4.2 ± 1.14	7.36 ± 4.72			
24	Chen K 2023	China	Bifidobacterium	5.71 ± 12.62	35	NA	NA	5.05 ± 0.48	3.4 ± 1.1	NA	1.0 × 10^10^	1	7
			Control	1.85 ± 6.34	35	NA	NA	5.56 ± 0.59	4 ± 1.3	NA			
25	Francavilla R 2012	Italy	Limosilactobacillus reuteri	26.1 ± 4.1	35	NA	19	2.1 ± 1.7	NA	NA	4.0 × 10^8^	1	7
			Placebo	25.4 ± 2.1	34	NA	9	3.3 ± 2.1	NA	NA			

CFU, colony forming unit.

### 3.3 Risk of bias assessment

**Figure 2:**
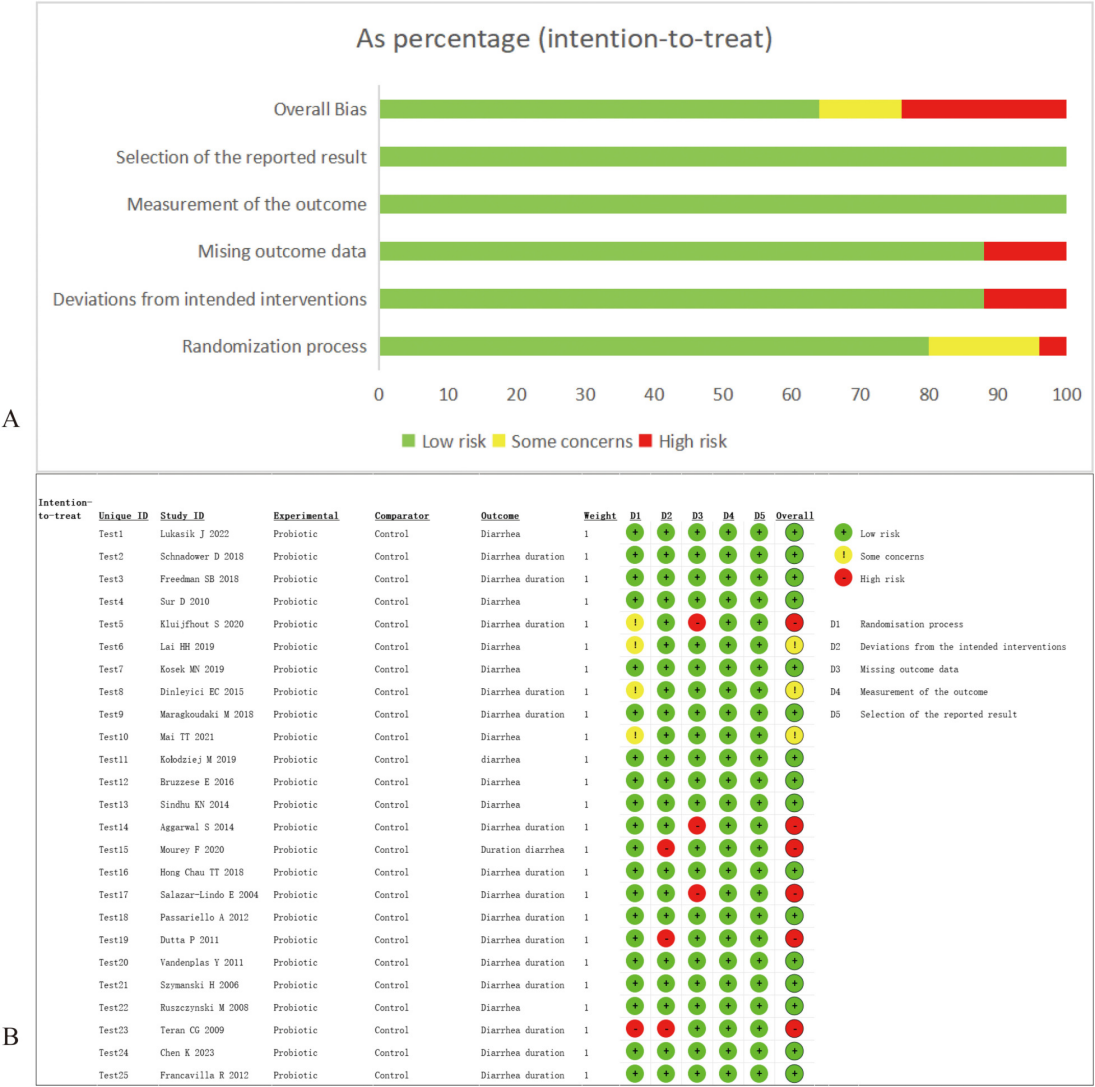
Results of risk of bias assessment. **(A)** Risk of bias graph: review authors’ assessments about each risk of bias item presented as percentages across all included studies. **(B)** Risk of bias summary: review authors’ judgments about each risk of bias item for each included study.

We assessed the risk of bias for the included studies. Of the 25 studies, 20 studies used an appropriate methodology for the process of randomization of participants, 22 studies did not deviate from the established interventions, 22 trials had complete outcome data, and the quality of the outcome measures and selective reporting was high for all RCTs. Nine trials (*n* = 1,848) were assessed as having a high risk of bias or some concerns, and 16 trials (*n* = 7,223) were assessed as having a low risk of bias (Figures 2A,B).

### 3.4 Outcome indicators

#### 3.4.1 Probiotics may shorten the duration of diarrhea in children

**Figure 3:**
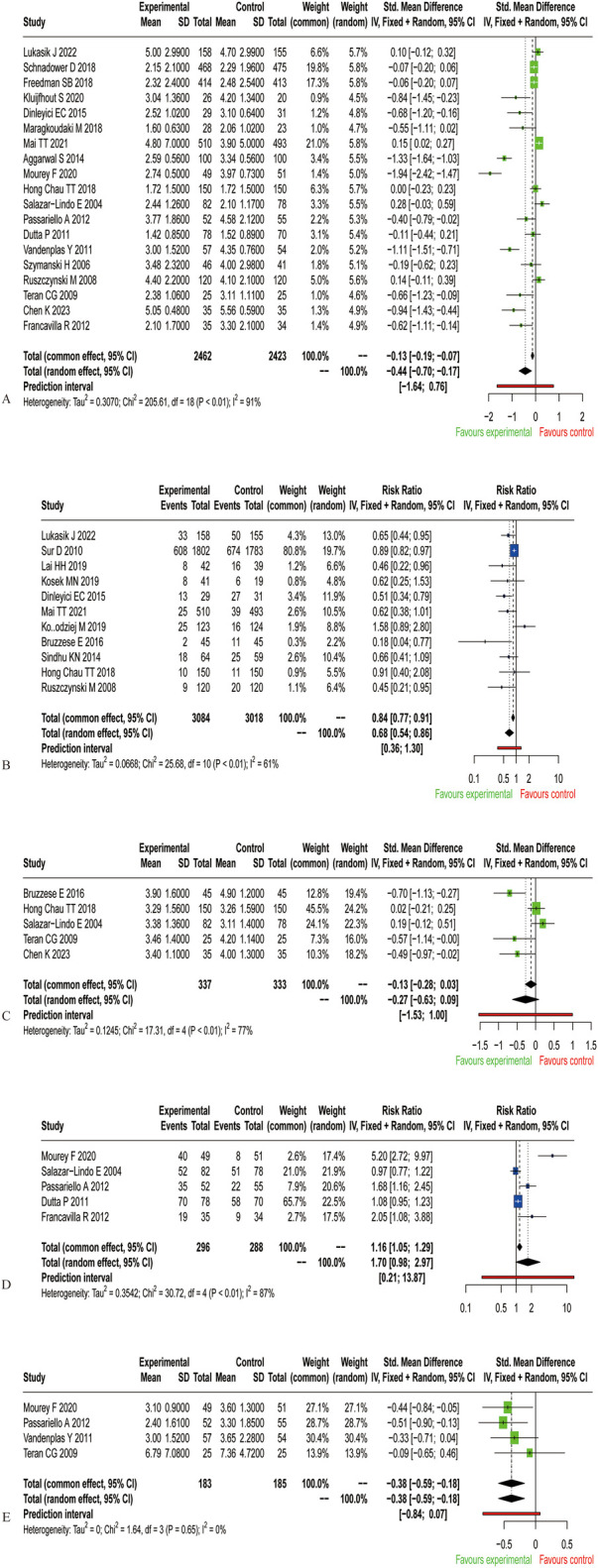
Results of a meta-analysis of probiotics for treating acute diarrhea in children. **(A)** Duration of diarrhea in children. **(B)** Number of children with diarrhea at the completion of the trial. **(C)** Length of hospitalization. **(D)** Number of recoveries within 3 d of intervention. **(E)** Frequency of stools on d 2.

To evaluate the effect of probiotics on diarrhea duration in children, we pooled data from 19 randomized controlled trials, involving 2,462 participants in the treatment group and 2,423 in the control group. Using a random-effects model, we found moderate-certainty evidence that probiotic supplementation significantly shortened the duration of diarrhea (SMD=−0.44, 95% CI: −0.70∼−0.17; Figure 3A and Supplementary Table S1), despite substantial heterogeneity among trials (I^2^ = 91%, *P* < 0.01). This corresponds to a reduction in the average diarrhea duration of approximately 1.21 days.

**Figure 4:**
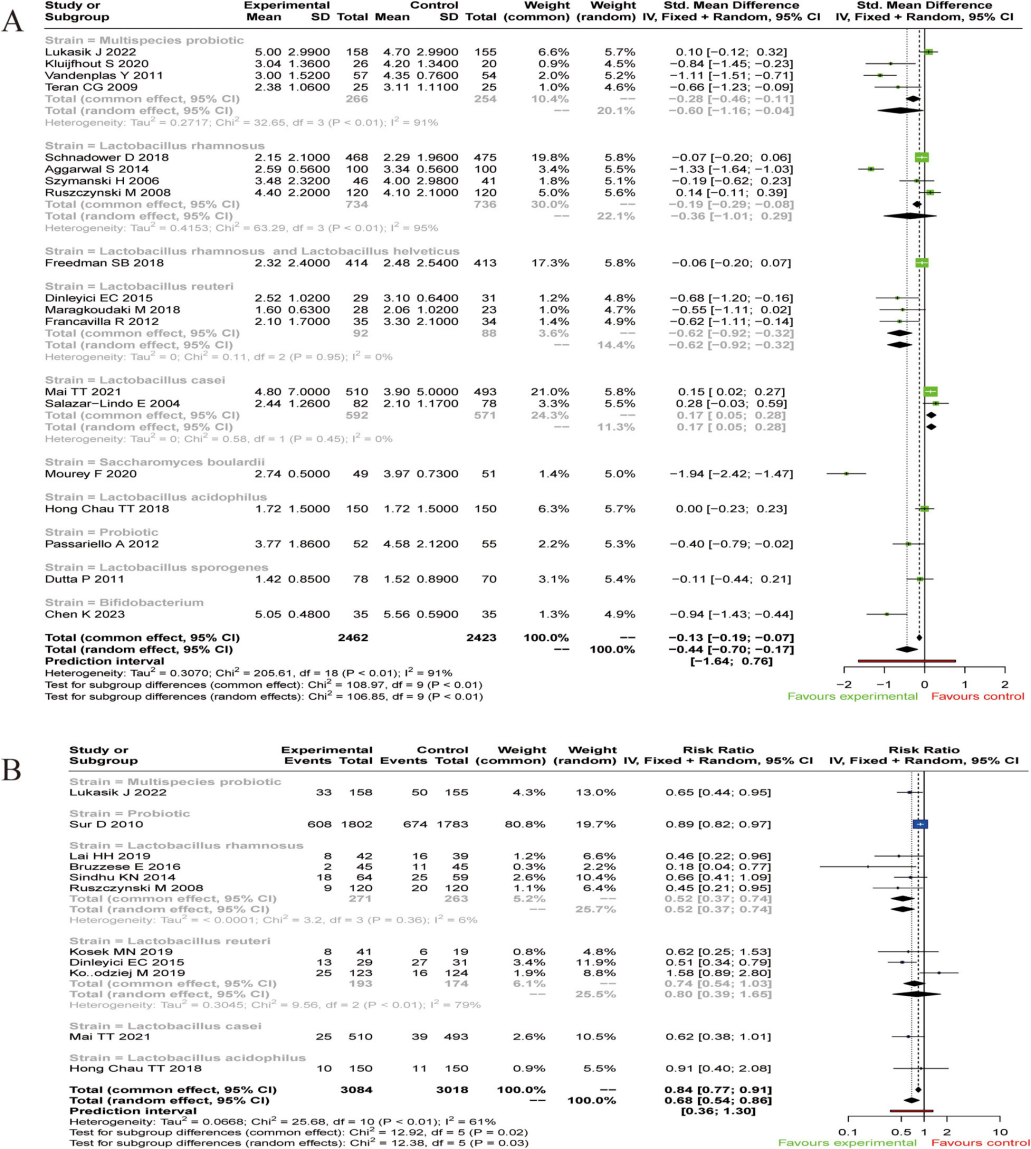
Subgroup analysis of primary outcomes. **(A)** Subgroup Analysis of Diarrhea Duration in Children. **(B)** Subgroup analysis of the number of children with diarrhea at the completion of the trial.

Subgroup analysis revealed that different strains exerted varying effects on the duration of diarrhea. Specifically, Multispecies probiotic (SMD=−0.60, 95% CI: −1.16∼−0.04), *Limosilactobacillus (L.) reuteri* (SMD = −0.62, 95% CI: −0.92∼−0.32), *Saccharomyces (S.) boulardii* (SMD = −1.94, 95% CI: −2.42∼−1.47), *Bifidobacterium* (SMD = −0.94, 95% CI: −1.43∼−0.44) significantly shortened the duration of diarrhea compared to placebo or no treatment. In contrast, no significant effect was observed for *Lacticaseibacillus (L.) rhamnosus* (SMD = −0.36, 95% CI: −1.01∼0.29), *L. rhamnosus* and *Lactobacillus (L.) helveticus* (SMD = −0.06, 95% CI: −0.20∼0.07), *Bacillus (B.) coagulans* (SMD = −0.11, 95% CI: −0.44∼0.21). Notably, *Lacticaseibacillus (L.) casei* was associated with a prolongation of diarrhea (SMD = 0.17, 95% CI: 0.05∼0.28; Figure 4A).

#### 3.4.2 Probiotics reduced the number of children with diarrhea at the completion of the trial and increased the number of children who recovered

To assess the effect of probiotics on recovery rates, we analyzed the proportion of children who still had diarrhea at trial completion. Eleven RCTs were included in the study, including 3,084 people assigned to the treatment group and 3,018 people assigned to the control group. Using a random-effects model, we observed that significantly fewer children in the probiotic group had persistent diarrhea at the end of the trial compared to the control group (RR = 0.68, 95% CI: 0.54∼0.86; Figure 3B). The evidence for this outcome was of moderate certainty (Supplementary Table S1), despite substantial heterogeneity among trials (I^2^ = 61%, *P* < 0.01).

Subgroup analyses revealed that the effect on recovery rates varied by probiotic type. Compared to placebo or no treatment, Multispecies probiotic (RR = 0.65, 95% CI: 0.44∼0.95), Probiotic (RR = 0.89, 95% CI: 0.82∼0.97), *L. rhamnosus* (RR = 0.52, 95% CI: 0.37∼0.74), significantly reduced the number of children with diarrhea at trial completion and increased the number of children who recovered. In contrast, no significant benefit was observed for *L. reuteri* (RR = 0.80, 95% CI: 0.39∼1.65) or *L. casei* (RR = 0.62, 95% CI: 0.38∼1.01; Figure 4B).

#### 3.4.3 Probiotics do not shorten hospital stays in children

To determine whether probiotics shortened hospital length of stay in children, five studies were included in the analysis of the effect of probiotics on hospital length of stay, including 337 patients assigned to the treatment group and 333 patients assigned to the control group. A pooled analysis using a random-effects model revealed no significant reduction in hospital length of stay associated with probiotic supplementation (SMD=−0.27, 95% CI: −0.63∼0.09; Figure 3C), Substantial heterogeneity was observed across trials (I^2^ = 77%, *P* < 0.01). Although probiotics did not shorten hospitalization compared with controls, the certainty of this evidence was assessed as very low (Supplementary Table S1).

#### 3.4.4 Probiotics did not affect the number of children who recovered within 3 d of the intervention

To determine the number of children who recovered within 3 d of the probiotic intervention, we statistically analyzed the number of children who recovered within 3 d and included 584 patients in 5 studies. Using a random-effects model, statistical pooling of these data revealed that probiotics did not significantly differ from controls in terms of the number of recoveries within 3 d of intervention (RR = 1.70, 95% CI: 0.98∼2.97; Figure 3D), and the heterogeneity of these results was high (I^2^ = 87%, *P* < 0.01). There was no significant trend in the number of patients who recovered within 3 d of intervention with probiotics compared with those who received the placebo or no treatment, but the quality of evidence for this finding was very low (Supplementary Table S1).

#### 3.4.5 Probiotics shorten stool frequency on d 2 in children with diarrhea

To determine the effect of probiotics on stool frequency in children with diarrhea, we analyzed stool frequency on d 2 in children. In four studies, including 368 patients who received the intervention, researchers reported the number of stools on d 2 after the intervention. Using a fixed-effects model, a pooled analysis of the data from these studies showed that probiotics significantly reduced the number of stools on d 2 in children with diarrhea (SMD=−0.38, 95% CI:−0.59∼−0.18; Figure 3E), and there was no heterogeneity between trials (I^2^ = 0%, *P* = 0.65), and the quality of evidence was very low (Supplementary Table S1). The results showed that probiotics reduced the frequency of stools on d 2 in the children compared with the control group.

## 4 Discussion

Diarrhea not only seriously jeopardizes the long-term physical development and health of children but also causes a large socioeconomic burden (42). To evaluate the clinical efficacy of probiotics in treating childhood diarrhea, we analyzed 25 randomized controlled trials involving 9,071 children. Our primary outcome, diarrhea duration, was assessed in 19 trials. The pooled results indicated that probiotic supplementation significantly shortened diarrhea duration (SMD=−0.44, 95% CI: −0.70∼−0.17), corresponding to a reduction of approximately 1.21 days, with moderate-certainty evidence.

We also evaluated recovery status at trial completion in 11 studies. Probiotics significantly increased the number of children who recovered (RR = 0.68, 95% CI: 0.54∼0.86), an outcome supported by moderate-certainty evidence that has been less frequently reported in prior research. Overall, probiotics showed beneficial effects on shortening diarrhea duration, promoting recovery, and reducing stool frequency compared to control. However, no significant effects were observed on hospital length of stay or on recovery within 3 days post-intervention. Notably, considerable heterogeneity across studies was observed, which limits confidence in the effect estimates.

Recent years have seen accumulating evidence supporting the antidiarrheal effect of probiotics in children with acute diarrhea. A previous Cochrane review demonstrated that probiotics significantly reduced the risk of diarrhea lasting >48 h (36 studies, *n* = 6,053, RR = 0.64, 95% CI: 0.52∼0.79) and shortened its mean duration (56 studies, *n* = 9,138, MD = −21.3 h, 95% CI: −26.9∼−15.7), despite substantial heterogeneity among included studies (43). Similarly, another meta-analysis on probiotics and synbiotics reported a reduction in diarrhea duration (28 studies, *n* = 3,883, WMD=−16.63, 95% CI: −20.16∼−12.51) (4). Both studies also indicated a potential benefit in shortening hospital stay (4, 43). However, our analysis did not find a significant effect of probiotics on hospitalization length. This discrepancy may be attributable to the high degree of heterogeneity observed across studies in this field.

Previous studies have reported that specific probiotic strains, including *S. boulardii* (MD = −0.66, 95% CI: −1.1∼−0.23), *L. rhamnosus* (MD = −0.66, 95% CI: −1.2∼−0.14), and *L. reuteri* (MD = −1.5, 95% CI: −2.3∼−0.61), reduce stool frequency on day 2 (44). Similarly, probiotics and synbiotics have been shown to decrease stool frequency on day 3 in children with acute diarrhea (7 studies, *n* = 1,040, WMD=−0.98, 95% CI: −1.55∼−0.40) (4). These findings align with our results, which indicate a reduction in stool frequency on day 2 with probiotic supplementation.

However, our findings diverge from prior research regarding early recovery. One study reported that probiotics significantly increased the 3-day recovery rate compared to control (RR = 0.59, 95% CI: 0.48∼0.73) (4). In contrast, our analysis did not demonstrate a significant benefit of probiotics on the number of recoveries within 3 days post-intervention.

In our analysis, subgroup analyses showed the effect of different probiotic strains on diarrhea in children. *L. reuteri* was associated with a significant reduction in diarrhea duration, whereas *L. rhamnosus* significantly reduced the number of children with diarrhea at the end of the trial. Furthermore, the multispecies probiotic demonstrated significant differences in both reducing the duration of diarrhea and increasing the number of recoveries. A recent study showed that *L. reuteri* reduced diarrhea duration (4 studies, *n* = 347, MD = −0.87, 95% CI: −1.43∼−0.31) (45). One more study also demonstrated that *L. reuteri* reduced the duration of diarrhea (MD = −0.84, 95% CI: −1.39∼−0.29) (44). In contrast, data analysis of another randomized controlled trial showed that *L. rhamnosus* had no effect on diarrhea duration (MD = −0.68, 95% CI: −1.81∼0.44) (46). One study reported that *L. rhamnosus* reduced the number of children with diarrhea (2 studies, *n* = 823, RR = 0.4, 95% CI: 0.2∼0.6) (47). These results are consistent with our conclusions.

Probiotics improve the prognosis of acute gastroenteritis in children through several key mechanisms: restoring intestinal microbiota balance, enhancing host immunity, and strengthening the gut barrier (8–12).

Firstly, probiotics promote the growth of beneficial gut microbiota by providing metabolites such as acetate, lactate, and propionate (48–50). Additionally, probiotics can directly impact the abundance of pathogens through a decrease in pH resulting from the production of lactate and short-chain fatty acids (SCFA), niche competition, or through bacteriocins (51–53). Furthermore, certain strains indirectly influence the resident microbiota by interacting with host epithelial cells and the epithelial immune system (10, 54, 55).

Secondly, probiotics influence immune regulation by modulating the expression of immune-related genes, inflammatory pathways, and key immune markers. Including modulation of intestinal epithelial cell nuclear factor kappa-light-chain-enhancer of activated B cells (NF-*κ*B), mitogen-activated protein kinase (MAPK), phosphoinositide 3-kinase (PI3K), peroxisome proliferator-activated receptor-*γ*, C-reactive protein (CRP), interleukin (IL)-6, IL-8, tumor necrosis factor (TNF)-*α*, IL-1β and interferon *γ* (IFN-*γ*) (10, 56).

Thirdly, probiotics can enhance the expression of tight junction proteins while stimulating goblet cells and epithelial cells to produce and secrete mucins and defensins, forming an immune barrier that prevents pathogenic bacteria from invading (57–59). Two secreted proteins purified from *L. rhamnosus* (termed p40 and p75) have been suggested to promote intestinal epithelial homeostasis by inhibiting cytokine-induced epithelial cell apoptosis (60). In summary, probiotics not only inhibit the overgrowth of pathogens but also enhance resistance to pathogenic microorganisms associated with diarrhea.

Our current study included a large number of RCTs and was reported in accordance with the PRISMA statement. The majority of included studies were assessed as low risk using the Cochrane Risk of Bias Assessment Tool. We also performed subgroup analyses to explorethe effects of different probiotic strains on childhood diarrhea.

Our study has several limitations. First, the number of available trials was limited for certain outcomes, which constrained the statistical power of the corresponding analyses. Second, the inclusion of studies with unclear or high risk of bias due to the scarcity of trials may affect the reliability of the pooled results. Third, mean values and standard deviations were not reported in several studies; we therefore derived these estimates from medians, quartiles, ranges, and sample sizes (61–64), a process that may have introduced inaccuracies. Fourth, long-term outcomes of diarrhea (growth retardation, malnutrition, and impaired cognitive development) were not assessed because few studies reported these outcomes. Fifth, substantial heterogeneity was observed across studies, likely attributable to variations in geographic settings, intervention protocols, and probiotic strains. Finally, the effect of different probiotic doses could not be examined due to insufficient data.

## 5 Conclusions

We conclude that probiotics are significantly effective in reducing the duration of diarrhea, increasing the number of recoveries in children with diarrhea, reducing the number of cases of diarrhea, and reducing diarrhea symptoms compared to placebo or no treatment.

## Data Availability

The original contributions presented in the study are included in the article/Supplementary Material, further inquiries can be directed to the corresponding author.
