# Supplementary material for: Probiotics for treating acute diarrhea in children: an evidence synthesis
Source: Front Pediatr. 2026 Jan 12;13:1722257. doi: 10.3389/fped.2025.1722257 (PMC12832844; doi:10.3389/fped.2025.1722257)
Supplement: Supplementary file 1 [file Datasheet1.docx]

**Supplementary Materials**

**SUPPLEMENTARY APPENDIX**

**Appendix 1.** Search strategies

**Table S1.** Certainty of evidence and summary effect estimates assessed by GRADE (grading of recommendations, assessment, development, and evaluation) of the study outcomes.

**Appendix 1.** Search strategies

We searched EMBASE, MEDLINE, PubMed, the Cochrane Library database, and the Clinicaltrials.gov registry. using a combination of MeSH and free text. The search terms include: diarrhea, probiotic, children, and randomized controlled trial. We did not add randomized controlled trial to search in the Clinicaltrials.gov registry.

| Search terms related to children | Child; Children |
| --- | --- |
| Search terms related to randomized  controlled trial | Randomized controlled trial; randomized; placebo; RCT |
| Search terms related to diarrhea | Diarrhea; diarrheas; diarrhea; diarrheas; acute diarrhea; diarrh*; gastroenteritis; gastroenteritides; acute gastroenteritis |
| Search terms related to probiotic | Probiotic; probiotics; probiotic*; prebiotics; prebiotic; prebiotic*; bifidobacterial; bifidobacterium; lactobacillaceae; lactobacilli; lactobacillus; saccharomyces; saccharomyces; saccharomyc*; saccharomyces boulardii; lactobacillus; enterococcus; escherichia coli; streptococcus; lactococcus; bacillus; lactobacilli; lactobacillus rhamnosus GG; lactobacillus reuteri; lactobacillus sporogenes; bifidobacterium longum; bifidobacterium breve; bifidobacterium bifidum; bifidobacterium lactis; bacillus; bacillus clausii; lactobacill*; streptococcus thermophilus; streptococc*; lactococc*; bacillus subtilis; enterococcus; enterococcus faec*; leuconostoc; pediococc*; bulgarian bacillus; bacillus laterosporus; pediococcus acidilactici; lactis |

**Table S1.** Certainty of evidence and summary effect estimates assessed by GRADE (grading of recommendations, assessment, development, and evaluation) of the study outcomes.

| **Certainty assessment** | | | | | | | **N of patients** | | **Effect** | | **Certainty** | **Importance** |
| --- | --- | --- | --- | --- | --- | --- | --- | --- | --- | --- | --- | --- |
| **N of studies** | **Study design** | **Risk of bias** | **Inconsistency** | **Indirectness** | **Imprecision** | **Other considerations** | **probiotics** | **no probiotics** | **Relative (95% CI)** | **Absolute (95% CI)** |  |  |
| **Duration of diarrhea in children** | | | | | | | | | | | | |
| 19 | randomised trials | not serious | serious^a^ | not serious | not serious | none | 2462 | 2423 | - | SMD **0.44 SD lower** (0.7 lower to 0.17 lower) | ⨁⨁⨁◯ Moderate^a^ | CRITICAL |
| **Number of children with diarrhea** | | | | | | | | | | | | |
| 11 | randomised trials | not serious | serious^b^ | not serious | not serious | none | 759/3084 (24.6%) | 895/3018 (29.7%) | **RR 0.68** (0.54 to 0.86) | **95 fewer per 1,000** (from 136 fewer to 42 fewer) | ⨁⨁⨁◯ Moderate^b^ | CRITICAL |
| **Length of hospitalization** | | | | | | | | | | | | |
| 5 | randomised trials | not serious | serious^c^ | serious^d^ | very serious^e^ | none | 337 | 333 | - | SMD **0.27 SD lower** (0.63 lower to 0.09 higher) | ⨁◯◯◯ Very low^c,d,e^ | IMPORTANT |
| **Number of recoveries within 3 days of intervention** | | | | | | | | | | | | |
| 5 | randomised trials | serious^f^ | serious^g^ | serious^d^ | very serious^e^ | none | 216/296 (73.0%) | 148/288 (51.4%) | **RR 1.70** (0.98 to 2.97) | **360 more per 1,000** (from 10 fewer to 1,000 more) | ⨁◯◯◯ Very low^d,e,f,g^ | IMPORTANT |
| **Frequency of stools on day 2** | | | | | | | | | | | | |
| 4 | randomised trials | serious^h^ | not serious | serious^i^ | very serious^e^ | none | 183 | 185 | - | SMD **0.38 SD lower** (0.59 lower to 0.18 lower) | ⨁◯◯◯ Very low^e,h,i^ | IMPORTANT |

**CI:** confidence interval; **RR:** risk ratio; **SMD:** standardised mean difference

#### Explanations

a. Serious inconsistency: I²=91%. There is significant heterogeneity between trials.

b. Serious inconsistency: I²=61%. There is significant heterogeneity between trials.

c. Serious inconsistency: I²=77%. There is significant heterogeneity between trials.

d. Serious indirectness: Only five studies reported this result. Further research may be needed before the results can be confidently generalized to all situations.

e. Serious imprecision: The 95% CI for the overall effect is wide, and the sample size is small.

f. Serious study limitations: Most studies are at high risk of bias.

g. Serious inconsistency: I²=87%. There is significant heterogeneity between trials.

h. Serious study limitations: Teran CG 2009 is not a randomization process.

i. Serious indirectness: Only four studies reported this result. Further research may be needed before the study results can be confidently generalized to all situations.
